# Quantifying lens obstructions in minimally invasive surgery: the impact on performance and outcomes

**DOI:** 10.3389/fsurg.2025.1576422

**Published:** 2025-04-09

**Authors:** Maciej Łącki, Megha Kalia, Nidhi Abraham, Sukesh Adiga Vasudeva, Dicken S. C. Ko, Timothée Bernard, Amy Lorincz

**Affiliations:** ^1^ Department of Research and Development, vopemed, Montreal, QC, Canada; ^2^ Department of Surgery, The Warren Alpert Medical School of Brown University, Providence, RI, United States

**Keywords:** surgical visualization, lens occlusion, lens fogging, laparoscopy, lens cleaning, surgical performance, operating time, robot assisted surgery

## Abstract

Surgeons performing laparoscopic surgery depend primarily on their vision to operate, but it often gets obstructed by fog, smoke, and other debris. This mini-review examines the literature on lens obstruction, aiming to quantify its prevalence, identify factors affecting its frequency, evaluate its impacts on surgeons and patients, and present an overview of mitigation methods. The review reveals that there are typically between 3.5–15 lens obstruction events per procedure, and surgeons spend between 19% and 52% of the procedure with suboptimal vision. Additionally, 2% to 8% of the operating time is devoted to cleaning the scope. Factors influencing the frequency of lens obstructions include instrument selection, operating time, and surgeon experience. Lens obstructions may increase operating time, the risk of medical errors, and mental fatigue, though quantifiable results on this subject remain sparse. The review also highlights significant knowledge gaps in the field of lens obstructions during minimally invasive procedures and proposes several recommendations to accelerate research in this area.

## 1 Introduction

Minimally invasive surgery (MIS) has revolutionized modern surgery by allowing surgeons to perform complex surgeries through small incisions, thereby reducing patient pain, scarring, complications, recovery times, and blood loss (1, 2). These procedures are performed using surgical instruments that lack tactile feedback. Consequently, surgeons must rely solely on a camera to visualize the surgical site (3). Unlike traditional open surgery, where surgeons directly view the operating site, laparoscopic surgeons rely on a small camera that can quickly become obscured by fog, smoke, or organic matter such as fluid and debris.

**Figure 1:**
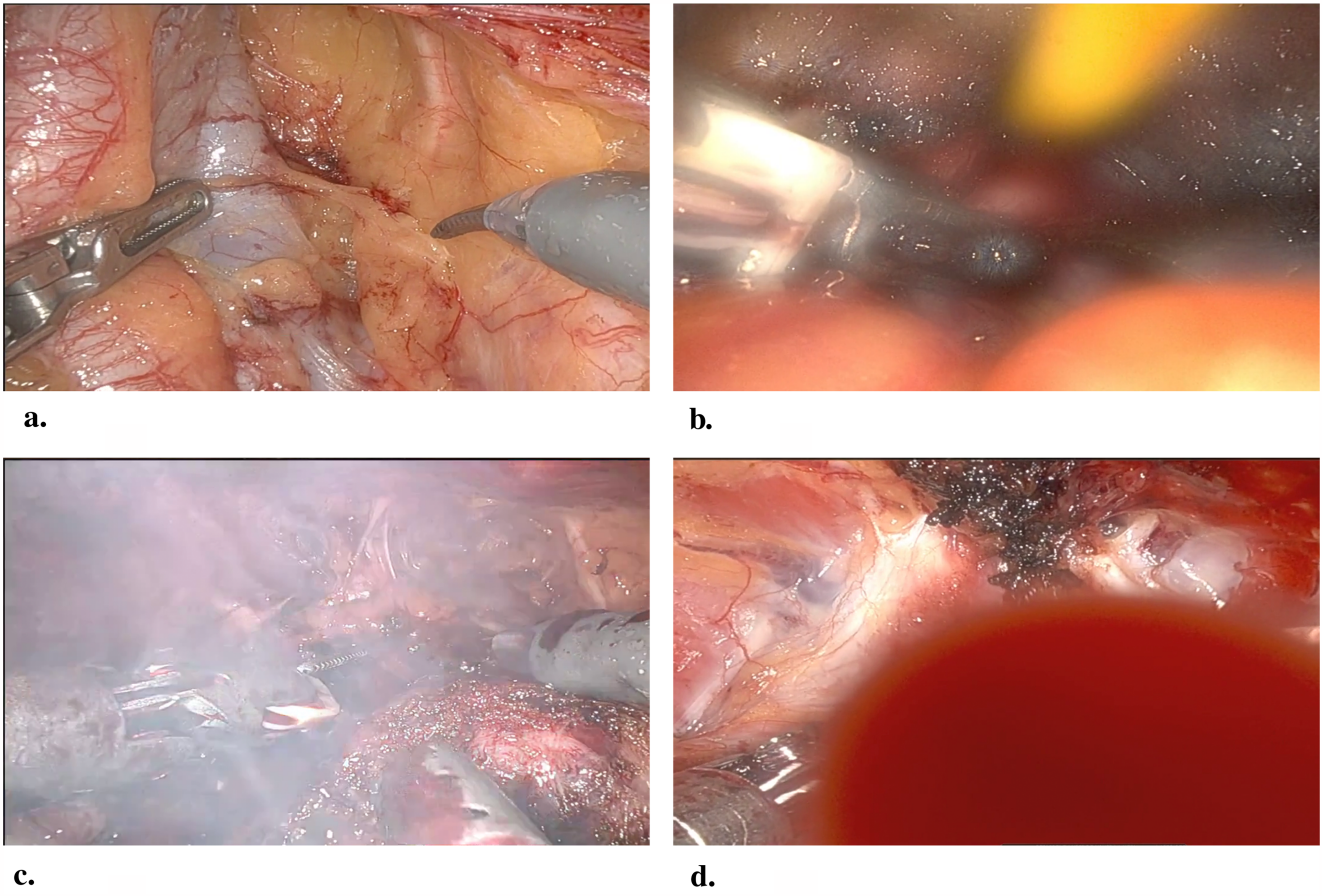
Clean lens **(a)**, fog **(b)**, smoke **(c)**, and fluid **(d)** observed during a robot-assisted radical prostatectomy procedure. Image credit: vopemed.

Figure 1 depicts the three lens obstructions: fog, smoke, and organic matter. Fogging occurs when water vapor condenses on the lens of a laparoscope (4). Specifically, this condensation happens when a surgeon inserts a room-temperature laparoscope into the patient’s warm and humid body cavity. On the other hand, smoke is generated by energy-based surgical devices (ESDs) like monopolar forceps. These devices are used to cut or burn tissue by locally applying heat. As a result, heating the tissue generates water vapor in the form of smoke plumes obstructing the surgeon’s view (5, 6). Additionally, organic matter such as blood, mucus, tissue fragments, and other bodily fluids (debris thereafter) physically stick to the lens. Frequent obstructions during laparoscopic procedures are a common source of frustration for surgeons (7–12), potentially endangering patient safety (7, 8). Although surgeons have various tools to manage these obstructions, they often need to interrupt their surgical workflow by removing the scope to clean the lens physically, diverting their attention away from the patient. In critical cases, surgeons may proceed with a dirty scope to urgently stop bleeding or switch to open surgery if maintaining clear vision becomes too difficult (13–15).

While the mechanisms of lens obstruction are well understood, their frequency, impact, and contributing factors remain unclear. This mini-review quantifies the frequency of lens obstructions, examines factors affecting their frequency and their impacts, as well as discussing the current mitigation methods. Additionally, the review identifies and highlights major research gaps and suggests improvements to study designs that could enhance our understanding of lens obstructions and their impacts.

## 2 Understanding lens obstruction

From the early days of laparoscopy, lens obstruction has posed significant challenges for surgeons (3, 7). Initially, it was studied as part of flow disruptions and distractions during operations (16–18). These studies bundled lens obstruction into broad categories like instrument troubleshooting, making it impossible to get any meaningful insight. This section reviews the findings of recent quantitative studies that specifically investigate lens obstructions and presents the current consensus, or lack thereof, on their factors and impacts.

### 2.1 Frequency of lens obstruction

Table 1 summarizes the quantifiable results of all studies that investigated lens obstruction in minimally invasive procedures. All but one study follow similar protocols except for Venkatayogi et al. who report the average time spent cleaning the lens for only 15 out of 28 analyzed procedures. Additionally, they used the Clearify Visualization System by Medtronic which means that the reported cleaning times do not represent nominal cleaning time (21).

**Table 1:** Summary of quantitative investigations into lens obstructions in minimally invasive surgery.

Study	Specialty	Surgical technique	Total number of observed procedures	Total procedure time (mins)	Average procedure time (mins)	Number of obstructions per procedure	Time spent operating with suboptimal vision (%)	Time spent cleaning the lens (%)
Yong et al. (19)	General surgery, gynecology, urology, general pediatric surgery	Laparoscopic surgery	25	3,830	154	15	37	7
Rahman et al. (20)	General surgery	Laparoscopic surgery	70	3,193	43.8	4.1	31.2	7.9
Nabeel et al. (9)	Bariatric surgery	Laparoscopic surgery	81	5,683	70	5.8	19.3	2.5
Venkatayogi et al. (21)	General surgery, gynecology, urology	Robot-assisted laparoscopy	28	2,660	95	5.8	52.5	2.2^a,b^
Dae et al. (15)	General surgery, gynecology, urology	Robot-assisted laparoscopy	45	5,696	127	3.5	41.4	1.2

^a^Cleaned the lens using Medtronic Clearify.

^b^Calculated based on 15 of 28 observed procedures.

The results indicate that on average surgeons encounter 3.5 to 5.8 obstructions per procedure and they spend 19.3% to 41.4% of the operating time with suboptimal vision. Additionally, surgeons must spend 1.2% to 7.9% of the operating time cleaning their scopes. Among these studies, Rahman et al. provide the only breakdown of types of obstruction encountered during their study with cautery, i.e., smoke, responsible for 53.2% of obstructions, condensation i.e., fogging, for 34.5%, and blood for 12.3% (20).

### 2.2 Factors affecting lens obstructions

The literature reports multiple factors that influence the frequency and severity of lens obstructions including the tools used during the procedure, procedure duration, and surgical team experience.

#### 2.2.1 Surgical instruments

The tools used during the procedure play a key role in the types, frequency, and severity of lens obstructions. For example, Yong et al. note that laparoscopes used in pediatric procedures have a smaller outer diameter, typically 4.9–6.0 mm, compared with 10 mm scopes used in adult procedures (19). A 1 mm^2^ piece of debris on a 10 mm scope obstructs only 1.3% of the area whereas the same debris on a 4.9 mm scope obstructs 5.3%, representing a 4-fold reduction of visibility.

On the other hand, Dae et al. report that unclear vision most commonly coincides with the use of cautery tools (37.9%) (15) which create plumes of smoke. The amount of smoke generated during the procedure depends on the tool type and the type of tissue. Monopolar forceps generate the most smoke, while bipolar forceps generate much less smoke, and harmonic cautery tools generate the least smoke (5, 6, 22). Additionally, cauterizing fatty tissue generates 17–23 times more smoke particles than lean tissue (23), which suggests that a patient’s body composition may also have an impact. Lastly, surgeons can control how much smoke they generate by setting the device power (24). Therefore, the amount of smoke generated during an operation depends on multiple factors of which tool selection and the tool power level are under the control of the surgeon.

#### 2.2.2 Operating time

Operating time is a likely factor, although there is no consensus on whether the relationship represents causation. Both Nabeel et al. (9) and Dae et al. (15) observe that longer procedures tend to encounter more obstructions. Nabeel et al. demonstrate a moderate positive correlation between total procedure time and time spent with compromised vision, total lens contamination events, and total cleaning events (9). Moreover, Dae et al. observe that urology procedures encounter more lens obstructions than other specialties since urology procedures are longer with more opportunities for the lens to get soiled (15). On the other hand, Abbitt et al. suggests that procedure complexity may not predict lens obstruction frequency (25), as they noted large variations in procedure time between similar procedures. Furthermore, Venkatayogi et al. did not find a statistically significant relationship between total procedure time and the number of lens obstructions (21).

#### 2.2.3 Surgical team experience

The surgical team experience also contributes to the frequency of lens obstructions. Interviews presented by Dae et al. suggest that the experience of both the technician and the surgeon plays a major role in maintaining clear vision (15). Furthermore, a pre-print by Ito et al. suggests that experienced surgeons skillfully avoid situations that risk soiling the lens, while experienced technicians more efficiently clean the lens (26). Surgeon’s experience is a complement factor that currently lacks quantitative evidence. While the argument is intuitively reasonable, further research should focus on validating this connection and examining the techniques that experienced surgeons use to minimize obstructions.

### 2.3 Overview of lens cleaning methods

Surgeons exercise a level of control on the frequency of lens obstructions. There are four general methods used to mitigate visual obstructions: physically wiping the scope, anti-fog solutions, scope warmers, and specialized equipment.

The simplest technique involves wiping the lens by withdrawing the scope and cleaning it with a cloth (19). This method disrupts the surgical workflow, leading some surgeons to wipe the obstructed lens on viscera, avoiding the disruption. However, the light attached to the endoscope may become hot enough to burn the patient’s organs (4, 27). Consequently, this method is falling out of favor with surgeons (9, 19). This technique eliminates obstructions but does nothing to prevent further obstructions from forming.

On the other hand, anti-fog solutions do not eliminate obstructions but attempt to prevent fog formation. Most anti-fog solutions are surfactants that produce a thin transparent film that reduces the surface tension, when applied to the lens. Reducing surface tension reduces the likelihood of condensation forming on the lens (28). These compounds may need to be reapplied during the operation, as the film is fragile and can be disrupted by other obstructions or cleaning tools. Surfactant use is widely supported, but its effectiveness varies, and there is a lack of quantitative data proving its capabilities (29).

Scope warmers prevent fogging by increasing the lens temperature above the dew point of the intra-abdominopelvic environment. The simplest form of scope warmer is a warm saline bath, but dedicated devices for this purpose also exist. Naturally, as the procedure progresses, the lens cools down, allowing fog to form once again, which means that scopes need to be warmed again. As a result, scope warmers disrupt the surgical flow (28).

In contrast, some specialized equipment can eliminate and prevent fog and other types of obstructions without requiring the withdrawal of the scope. For instance, insufflation systems take the form of a modified laparoscope with a channel that allows ${\text{CO}}_{2}$ gas to flow to the tip of the lens and remove fogging and other types of obstructions. These systems can mitigate most obstructions without the need to withdraw the instrument, but they are bulkier and require the use of a 12 mm trocar, which may not be feasible for all laparoscopic procedures (28).

An in-depth review of the obstruction mitigation methods revealed that there is not enough evidence to identify any single method as superior to others (28) and that they do not have a significant impact on procedure outcomes compared to the control (29).

### 2.4 Clinical impact of lens obstruction

Although widely reported as a possible source of complications and frustration, lens obstruction’s full clinical impact remains unquantified due to a lack of comprehensive studies. This section examines the available direct and circumstantial evidence while highlighting critical research gaps.

#### 2.4.1 Postoperative complications

Cheng et al. show that even a small increase in operating time raises the likelihood of postoperative complications. For instance, 1 min increases the risk by 1% and 10 min by 4% (30). Based on Table 1, surgeons spend from 1.7 to 10.6 min per procedure cleaning their scopes, which raises the risk of complications by 1%–4%. However, the frequency and length of lens obstructions have a high variance (9, 15, 21). For instance, Nabeel et al. note that in the most severe case, surgeons spent 15 min cleaning their lens (9). Therefore, a 4% increase in the risk of complications represents a conservative estimate with the other factors further compounding the risk.

#### 2.4.2 Risk of errors

Operating without clear visibility further elevates the risk of errors. A survey of 109 surgeons reveals that 61% of surgeons witnessed surgical complications and errors resulting directly from lens obstructions, with 90% also stating that obstructed vision compromises patient safety (9). However, surgeons often choose to not interrupt the procedure and continue with an obstructed view (9, 15, 19), especially if they are performing a crucial part of the surgery, as it takes too long to clean the lens (15). Even though the survey results do not provide quantifiable data, they clearly highlight a need to minimize the time surgeons spend with an obstructed view. To achieve this, novel quick and less disruptive tools need to be developed to clean the lens.

#### 2.4.3 Mental fatigue

Additionally, lens obstructions may contribute to surgeons’ mental fatigue. Nabeel’s survey correlated obstructions with frustrations and measured the surgeons’ perceived workload scores. On average, surgeons scored 71.7/100 on the National Aeronautics and Space Administration Task Load Index (NASA-TLX), with mental demand and frustration scoring the highest (9). An increase in NASA-TLX scores has been shown to predict surgeon performance, specifically the risk of injuries to patients (31) with scores exceeding 50/100 elevating the risk of medical errors (32, 33).

## 3 Discussion

Despite overwhelming anecdotal evidence, surgeon surveys, and general intuition that lens obstruction should increase the surgery duration, deteriorate surgeon performance, and increase injury risks to patients, quantifying the phenomenon remains elusive (7–12). There are two possible explanations, either lens obstruction has only a negligible impact on surgeon performance and the patient outcomes, or the currently available lens cleaning tools are too disruptive and require too much time to operate causing the surgeons to not clean their camera lens (29). Given the inability to measure surgeon performance and patient outcomes without lens obstructions, and considering surgeons’ hesitancy to sacrifice surgical workflow for better visibility (15), the latter explanation seems more plausible.

Even though difficult, it could be possible to estimate the impacts of lens obstruction given a large enough structured set of data. Though rare, in almost every quantitative study, a handful of outlier procedures encountered no lens obstructions. With a large enough sample size, a statistical meta-analysis could estimate the duration of a procedure in ideal conditions along with the ideal patient outcomes. However, even with more studies the current reporting methods make such an analysis impossible. For instance, (15, 19, 21) assess a variety of specialties and procedures with an average of about 10 cases per specialty. However, (9, 20) show that the procedure duration and number of obstructions vary widely even within a single procedure. Additionally, many sources do not distinguish different types of obstructions, with many researchers using the term, *laparoscopic lens fogging*, to refer to any type of obstruction (9). Studies, also, rarely report patient outcomes, which makes it difficult to evaluate how lens obstructions affect them. In summary, the studies performed thus far are small in scale and often fail to disclose granular data like types of obstructions, patient demographics, patient outcomes, and unintended tissue damage making it impossible to fully understand the frequency, factors, and impacts of lens obstructions.

As a result, a few improvements to study design are needed. Any study collecting data about interruptions and obstructions during a procedure needs to publish more granular statistics. At the very least, the data should include the specific procedure type, its duration, the number of contamination events, the duration of lens cleaning activities, and the amount of time spent with obstructed vision. Additionally, data on the approximate cause of the obstruction, the subjective surgeon experience including mental fatigue, as well as surgeon and team experience levels would help in quantifying the impact of lens obstruction on the surgeon. Furthermore, collecting patient de-identified clinical information, number of bleeding events, estimated blood loss, the number and severity of surgical errors, as well as the approximate amount of tissue damaged during the procedure would help to better understand how lens obstruction affects patient safety.

Unlike other factors, the role of smoke has been well scrutinized, and the results suggest that it does not have a major impact however, using tools that generate less smoke makes the procedure easier to perform but it does not affect its duration (6). Furthermore, lens obstructions resulting from tissue cauterization did not significantly increase the risk of complications or the duration of the procedure (6, 34). Nevertheless, smoke may increase the likelihood of fogging by raising the humidity inside the body cavity, however the link has yet to be shown (28). Clearly other forms of obstructions deserve evaluation with a similar level of scrutiny as, unlike smoke, surgeons have minimal control over when and how they occur.

### 3.1 Concluding remarks

Lens obstruction, including fogging, smoke, and physical debris, are widely reported as sources of frustration for laparoscopic surgeons (7–12), but there is limited research on the frequency and clinical impacts of these obstructions. This mini-review presents the research on the subject and highlights the contributing factors, consequences of lens obstructions on surgeons and patients, and current knowledge gaps. Quantitative analysis shows that during a procedure surgeons operate with suboptimal vision between 19.3% and 52.5% of total operating time and their view gets obstructed between 3.5 to 15 times. The frequency of obstructions depends on the type of procedure, tools, duration of the surgery, and surgeon and technician experience. The factors that affect the frequency and severity of lens obstructions include diameter of the laparoscopy, the type of electrocautery tool in use, the tissue composition of the patient, the operating time, and the surgical team experience. The evidence suggests that lens obstructions may increase the risk of postoperative complications by 1%–4% just based on the time spent cleaning the lens. Given that the majority of surgeons believe that lens obstructions compromise patient safety and report seeing medical errors resulting from them, the 1%–4% range is a conservative estimate and with more data the range could be further refined.

Notably, there are substantial gaps and issues in the way that lens obstruction is currently studied. There is a general lack of quantifiable data showing the impact of lens obstruction on surgeon performance and patient outcomes. The lack of data transparency and granularity makes it impossible to perform a thorough meta-analysis. Additionally, many studies fail to distinguish between different types of lens obstruction, and there is little research on the causes and factors that alleviate or exacerbate lens obstruction. As a result, it is difficult to determine the true impact of lens obstructions on surgeons’ performance and patient outcomes. Furthermore, the current mitigation methods lack quantitative assessments making it difficult to determine how effective they are. This manuscript proposes a range of study design improvements that would facilitate a more thorough understanding of lens obstructions.

In conclusion, this review affirms what surgeons worldwide intuitively understand: lens obstructions are common and negatively affect both their performance and patient outcomes (7–12). Despite the difficulty in quantifying these impacts, it is clear that surgeons seek more effective, less disruptive, and faster cleaning tools (15), including software solutions using machine learning (10, 12). Developing more robust tools would allow surgeons to maintain their surgical workflow without sacrificing their ability to see what they are doing. With such tools, surgeons could save time and operate under less stress, likely resulting in fewer injuries and complications for the patient. Marian Wright Edelman, an American civil rights activist, once said, *You can’t be what you can’t see*. Her words resonate beyond civil rights, because in surgery, *You can’t cut what you can’t see*.
